# Vegan Diet, Subnormal Vitamin B-12 Status and Cardiovascular Health

**DOI:** 10.3390/nu6083259

**Published:** 2014-08-19

**Authors:** Kam S. Woo, Timothy C.Y. Kwok, David S. Celermajer

**Affiliations:** 1 Room 186, Science Centre South Block, Biochemistry Programme, School of Life Sciences, The Chinese University of Hong Kong, Shatin NT, Hong Kong; 2 Department of Medicine and Therapeutics, The Chinese University of Hong Kong, Hong Kong; E-Mail: tkwok@cuhk.edu.hk; 3 Sydney Medical School, The University of Sydney, Sydney 2050, Australia; E-Mail: david.celermajer@email.cs.nsw.gov.au

**Keywords:** vegan diet, vitamin B-12 deficiency, atherosclerosis surrogates, cardiovascular health

## Abstract

Vegetarian diets have been associated with atherosclerosis protection, with healthier atherosclerosis risk profiles, as well as lower prevalence of, and mortality from, ischemic heart disease and stroke. However, there are few data concerning the possible cardiovascular effects of a vegan diet (with no meat, dairy or egg products). Vitamin B-12 deficiency is highly prevalent in vegetarians; this can be partially alleviated by taking dairy/egg products in lact-ovo-vegetarians. However, metabolic vitamin B-12 deficiency is highly prevalent in vegetarians in Australia, Germany, Italy and Austria, and in vegans (80%) in Hong Kong and India, where vegans rarely take vitamin B-12 fortified food or vitamin B-12 supplements. Similar deficiencies exist in northern Chinese rural communities consuming inadequate meat, egg or dairy products due to poverty or dietary habits. Vascular studies have demonstrated impaired arterial endothelial function and increased carotid intima-media thickness as atherosclerosis surrogates in such metabolic vitamin B-12 deficient populations, but not in lactovegetarians in China. Vitamin B-12 supplementation has a favourable impact on these vascular surrogates in Hong Kong vegans and in underprivileged communities in northern rural China. Regular monitoring of vitamin B-12 status is thus potentially beneficial for early detection and treatment of metabolic vitamin B-12 deficiency in vegans, and possibly for prevention of atherosclerosis-related diseases.

## 1. Introduction

Vegetarian diets have been recognized as potentially cardio-protective. Many people start vegetarian diets amounting to 5%–6% of population for perceived health benefits, emphasizing more fruits, vegetables, nuts and grains but less dairy or meat products [1,2,3,4]. There is sizeable literature to document a lower prevalence of diabetes mellitus, hypertension, hypercholesterolemia [5,6], lower prevalence and mortality from ischemic heart disease and stroke in vegetarians, as compared with non-vegetarian omnivores [5,6,7,8].

To (potentially) extend the health benefits, vegan diets (vegetarian diets without dairy or egg products) are increasingly popular, particular among young women (prevalence of 2%–4%). Preference of such nutritional choices centre around better care of earth resources and environment, ethical issues on animal care, use of growth stimulants and antibiotics for rearing of animals, concern about animal-borne diseases, potential allergies and lactose intolerance from dairy products. While evidence from epidemiology studies in the past two decades are suggesting that most balanced vegetarian diets are nutritionally adequate and associated with a lower risk of coronary artery disease and stroke [9,10,11], the advantage of strict vegan diet as compared with lactovegetarian diet remains unproven, and thus advocacy for vegan diets has been cautious [9,10].

The present paper aims to specifically address and review the existing evidence concerning the possible impact of vegan or related diets on cardiovascular health.

## 2. Methods

We performed an independent literature review via internet using PubMed/Medline, EMBASE Google and Yahoo database from 1997 to July 2014. The following search keywords were used: vegetarians/vegetarian diets, vegan, vegan diets, cardiometabolic risk, vitamin B-12 status, cardiovascular risk, cardiovascular disease or events, atherosclerosis surrogates. Inclusion criteria were: (a) prospective cohort studies or review of studies in either or both genders; (b) data comparing cardiovascular risk and outcome in vegetarians *vs.* non-vegetarians and/or vegans; (c) reports with well defined dietary patterns as exposures. Animal studies and studies not reporting relevant cardiovascular health or subclinical atherosclerosis outcomes were excluded. We adopted the definition of non-vegetarians as omnivores consuming red meat, poultry, fish, dairy or egg products regularly, lacto-ovo-vegetarians as those consuming dairy and egg products regularly, but no red meat, poultry or fish for over two years, and vegans as those consuming no red meat, dairy/egg products, poultry nor fish for over two years. Our review specifically focused on their vitamin B-12 status and cardiovascular risk or outcome.

## 3. Atherosclerosis Surrogate and Vascular Epidemiology

**Figure 1:**
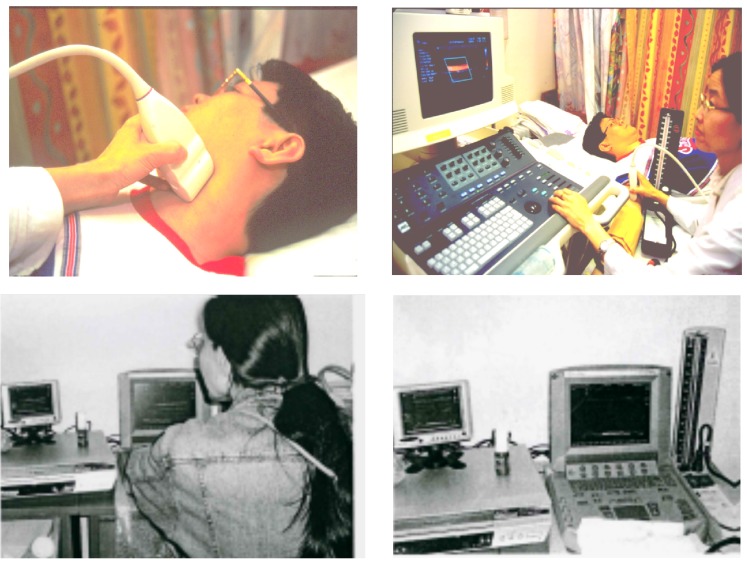
Ultrasound scan of carotid artery for carotid intima-media thickness and brachial endothelium-dependent dilation (endothelial function), using an ultrasound console (**above**); or a portable ultrasound machine at field work in northern China (**below**).

Epidemological studies on the prevalence of atherosclerosis-related disease in vegans are scarce. Those few studies available show that vegan diet in 21 sedentary subjects is associated with lower cardio metabolic risk (lower fasting glucose, insulin and lipid profiles and body mass index) compared with 21 sedentary subjects on traditional western diets [11], but studies of Adventist and EPIC-Oxford cohorts showed no definite protection of strict vegan against non-vegetarian life styles (hazard ratio of ischemic heart disease mortality 0.90 (0.60–1.33); cardiovascular disease mortality 0.91 (0.71–1.16)) [12,13]. Atherosclerosis surrogates may thus be useful, in the absence of clinical endpoint studies. In this regard, flow-mediated endothelium-dependent dilation (vascular reactivity, FMD) and carotid intima-media thickness (inner lining, IMT) have emerged as atherosclerosis surrogates in the past two decades (Figure 1) [14,15,16,17]. Atherosclerosis begins early in life. Endothelial dysfunction contributes to atherogenesis and precedes the development of vascular morphological changes. Measurement of endothelial function has thus given insights into early atherosclerosis in particular. Many studies have suggested that non-invasive ultrasound-derived endothelial function assessment using stringent protocols be reproducible and informative for cardiovascular risk and assessment of overall atherosclerosis burden [18,19,20,21,22,23]. For instance, endothelial dysfunction is associated with traditional atherosclerosis risk factors (active and passive smoking, hypercholesterolemia, hypertension, obesity and diabetes mellitus), hyperhomocysteinemia and coronary artery disease [24,25,26,27,28,29,30], with improvement on correction of these risk factors, in certain circumstances [31,32]. Moreover, using FMD as a marker of endothelial dysfunction, Chinese adults were documented to be less susceptible than whites to age-related endothelial dysfunction [33]; young Chinese adults have less endothelial dysfunction than white adults with similar exposure to active or passive cigarette smoking [34]; westernized Chinese in Sydney and San Francisco are more susceptible than southern rural Chinese to dose-related vascular effects of smoking and to the deleterious impact of low high-density lipoprotein cholesterol levels [35].

Similarly, carotid IMT derived by high resolution ultrasound is highly reproducible, correlates well with severity and extent of coronary artery disease [36,37], and may be predictive of stroke and coronary events in asymptomatic healthy subjects [16,17,38]. Increased carotid IMT has been shown to be a marker of subclinical atherosclerosis in westernized Chinese and in rural India [35,36,37,38,39], and is associated with a more atherogenic dietary pattern. Similar high susceptibility to risk factors for carotid atherosclerosis in populations undergoing rapid economic changes has been demonstrated in southern Asians and in Chinese immigrants to Canada [40]. Significant improvements in carotid IMT have been reported in coronary populations after adjunctive traditional Chinese medicine treatment [41], and in renal failure patients after high dose folic acid supplementation [42].

## 4. Metabolic Vitamin B-12 Deficiency with Vegan or Related Diets

Vegetarians and in particular vegans consume high amounts of fruits and vegetables, grains and nuts but their intake of vitamin B-12 is usually inadequate [43,44]. Subnormal vitamin B-12 status is prevalent (50%–70%) in vegetarians or vegans in Austria Germany, Italy, Australia, India and China [45,46,47,48,49,50,51,52]. There has been some controversy about the definition of vitamin B-12 deficiency because of variability in clinical manifestation. Metabolic deficiency, as evidenced by raised plasma homocysteine or methylmalonic acid, may be a more practical means of assessing compromised vitamin B-12 status. Serum vitamin B-12 levels less than 300 pmol/L are associated with a significant risk of metabolic vitamin B-12 deficiency in epidemiological studies [53]. Such metabolic vitamin B-12 deficiency was present in vegetarians/vegans in Australia, Germany, Taiwan, Hong Kong, Italy, and India [45,46,47,48,49,50,51,52]. Madry *et al*. have reported a significant decrease of serum vitamin B-12 from 285 pg/mL to 230 pg/mL (*p* < 0.0001) in omnivores changing to strict vegan diet for five years, consuming exclusively natural product, but not in those vegans consuming vitamin B-12 fortified products [54]. Regular intake of milk improved vitamin B-12 status and lowered blood homocysteine level in young Indian vegetarians [55], but has been avoided in most Chinese vegetarians. In studying the impact of poor intake of meat and dairy products on vitamin B-12 status in rural Chinese coalminers in Shanxi, Woo *et al.* have documented subnormal vitamin B-12 level (196 ± 104 pmol/L) in 207 coalminers, whereas vitamin B-12 levels were normal (477 ± 170 pmol/L) in southern Chinese omnivores consuming usual Chinese diets [56,57]. These data highlight the potentially detrimental impact of inadequate micronutrient intake on vitamin B-12 status.

## 5. Atherosclerosis Surrogates and Subnormal Vitamin B-12 Status

Toohey *et al.* reported that cardiovascular risk factors are more favourable in African-American vegans, compared with lacto-ovo-vegetarians, with significantly lower body mass index (24.7 ± 1.9 *vs.* 26.4 ± 0.45 kg/m^2^), lower total cholesterol (3.75 ± 0.12 *vs.* 4.51 ± 0.1 mmol/L), and lower low-density lipoprotein (LDL) cholesterol levels (2.06 ± 0.13 *vs.* 2.65 ± 0.09 mmol/L), *p* < 0.05 [58]. Specific vitamin B-12 and vascular surrogates data, however, were lacking. Su *et al.* have found significantly lower vitamin B-12 (273.4 ± 184.8 *vs.* 359.7 ± 138 pmol/L), higher homocysteine (10.8 ± 3.2 *vs.* 9.2 ± 2.2 umol/L) and sVCAM-1 (724.7 ± 418.3 *vs.* 547.5 ± 259.9 mg/mL), lower brachial artery resistance (4122 ± 1418 *vs.* 4977 ± 1936 mmHg/L/min), but similar carotid IMT, carotid beta stiffness, and brachial artery compliance in Chinese vegetarians (89% vegans) in Taiwan, compared with omnivores [59,60]. However apparently healthy lactovegetarian Chinese men aged 24–55 years were found to have lower cardiovascular risk factor levels (body mass index, systolic and diastolic blood pressure, lipid profile and insulin secretion index) than omnivores, associated with reduced carotid IMT [61].

In Chinese non-smoking vegetarians in Hong Kong, aged 45 ± 10 years, vascular dysfunction was demonstrated by Kwok *et al.* compared with age-matched non-smoking omnivores, with higher mean blood pressure (99.0 ± 13.2 *vs.* 93.7 ± 9.6 mmHg, *p* < 0.05) and plasma homocysteine (12.9 ± 7.8 *vs.* 9.2 ± 7.6 μmol/L, *p* < 0.005), lower vitamin B-12 (189 ± 16.5 *vs.* 337 ± 137.8 pmol/L, *p* < 0.005), higher blood folate (45 ± 22 *vs.* 36 ± 16 nmol/L, *p* < 0.05) and triglycerides (1.4 ± 1.4 *vs.* 0.9 ± 1.2 mmol/L, *p* < 0.05) [52]. Despite better total and LDL-cholesterol profiles, their mean carotid IMT was greater (0.69 ± 0.09 *vs.* 0.56 ± 0.11 mm, *p* < 0.0001) and brachial FMD was lower (6.4% ± 1.8% *vs.* 10.0% ± 2.6%, *p* < 0.0001) compared with omnivores. On multivariate regression analysis, age, male gender and vegetarian group were independently related to carotid IMT, whereas vegetarian group and triglyceride levels were independently correlated with lower FMD. Of note, to make the meal more tasteful, the consumption of salt and carbohydrates in Hong Kong Chinese vegetarians were relatively high, which could explain the higher mean blood pressure and triglycerides among the vegetarians [44]. In normotensive or hypertensive nonvegetarian adults, high sodium intake or urinary sodium excretion is associated with increased carotid intima-media thickness and alteration of carotid elastic modulus [62,63].

Abnormal vascular function and structure were also observed in 207 rural northern Chinese (38% smokers) with a mean-age of 48 ± 8 years. In association with metabolic vitamin B-12 deficiency, their carotid IMT (0.71 ± 2.15 mm), and brachial FMD (6.8% ± 7.4%) were significantly worse than age-matched southern Chinese (40% smokers) with normal vitamin B-12 status (0.56 + 0.11 mm and 10.0% ± 2.6% respectively) [56,57].

## 6. Impact of Vitamin B-12 Supplementation

Inadequate intake of vitamin B-12, is associated with a certain degree of metabolic B-12 deficiency. Consumption of dairy or egg products (lacto-ovo-vegetarian) may partially alleviate this. The impact of vitamin B-12 supplementation on vascular surrogates was reported by Kwok *et al.* [64]. For this, 50 healthy vegetarians with vitamin B-12 <150 pmol/L in 70%, were randomized in double blind cross-over design to receive vitamin B-12 (500 μg/day) or identical placebo capsules for 12 weeks before cross-over. Vitamin B-12 supplementation significantly increased serum vitamin B-12 level (134.0 ± 125.6 to 379.6 ± 206.2 pmol/L, *p* < 0.0001) and lowered plasma homocysteine (16.7 ± 11.0 *vs.* 11.3 ± 6.0 μmol/L, *p* < 0.01), associated with significant improvement of brachial FMD (6.3% ± 1.8% to 6.9% ± 1.9%, *p* < 0.0001), and carotid IMT (0.69 + 0.9 to 0.67 ± 0.9 mm, *p* <0.05). After subsequent open label vitamin B-12 treatment for additional 24 weeks, there were further improvement in brachial FMD (to 7.4% ± 1.7%, *p* <0.0001) and carotid IMT (to 0.65 ± 0.09mm, *p* <0.001). Changes in vitamin B-12 (β = 0.25, *p* = 0.02), but not homocysteine were related to changes in FMD (*R* = 0.32, *F* value = 3.19, *p* = 0.028).

**Figure 2:**
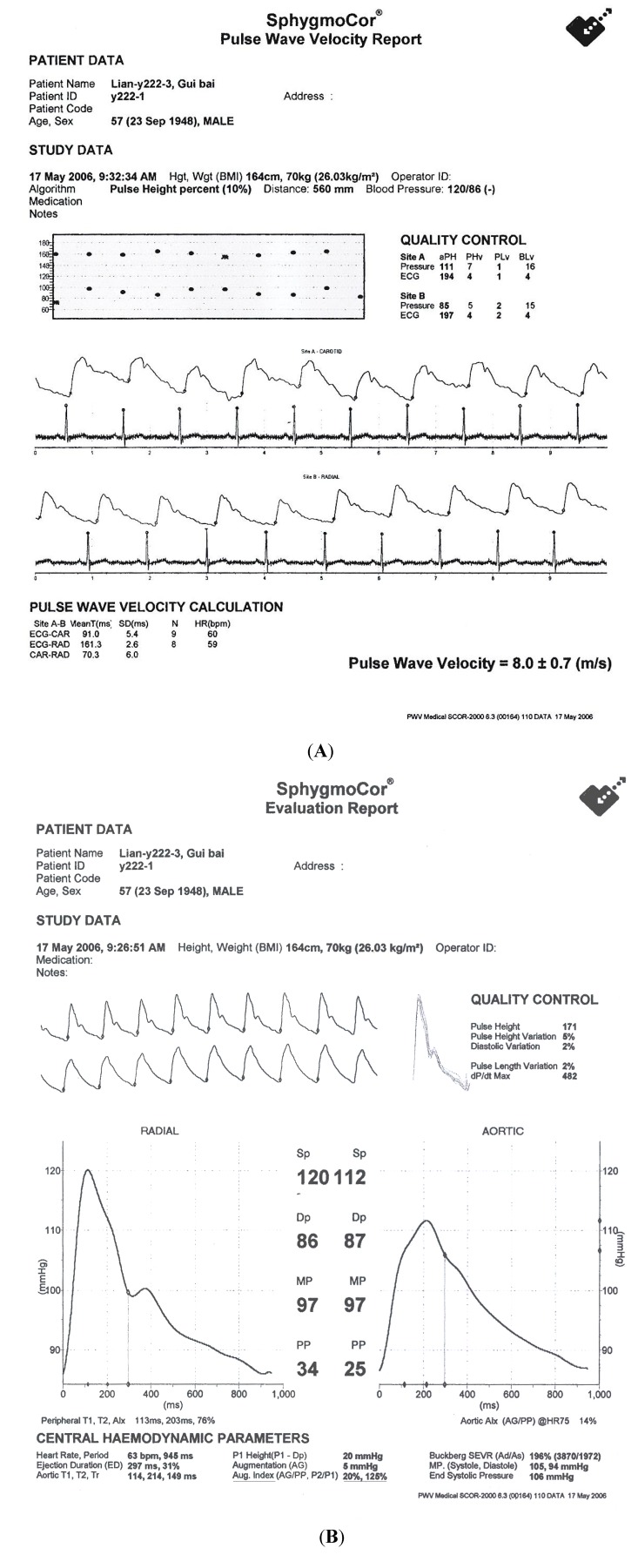
Measurement of pulse wave velocity (**A**) and aortic augmentation index (**B**) as marker of arterial stiffness by SphygmoCor machine (Atcor Medical, Sydney, Australia).

Further confirmation of the potential cardiovascular benefit of vitamin B-12 supplement in metabolic vitamin B-12 deficiency was reported by Woo *et al*. in a cohort of Shanxi coalminers (northern China) [56]. For this, 207 asymptomatic coalminers, aged 48+8 years, were randomized to receive oral vitamin B-12 (500 μg/day, *n* = 52), placebo (*n* = 53), folic acid (5mg/day, *n* = 51) and combination vitamin B-12 and folic acid supplementation (*n* = 51) for 6 months in a double blind trial. Serum vitamin B-12 significantly increased after vitamin B-12 (214 ± 10.9 to 305 ± 131 pmol/L, *p* < 0.001) or vitamin B-12 and folic acid combination treatment (188 ± 109 to 289 ± 170 pmol/L, *p* < 0.0001), but not after placebo, associated with a significant decrease in plasma homocysteine (24.5 + 13.7 to 18.8 + 10.5 μmol/L, *p* < 0.001) after folic acid or vitamin B-12/folic acid combination treatment (20.9 ± 10.9 to 15.1 ± 10.1 μmol/L, *p* < 0.001). Further improvement in brachial FMD (6.5% ± 1.4% to 8.2% ± 1.4%, *p* < 0.0001) and carotid IMT (0.71 ± 0.14 to 0.69 ± 1.14 mm, *p* < 0.005) were observed after subsequent open label folic acid/vitamin B-12 combination treatment, independent of homocysteine (*p* = 0.5) and blood pressure-lowering (*p* = 0.1). In addition significant reduction in systolic blood pressure were seen after folic acid and vitamin B-12/folic acid combination treatment, as well as diastolic blood pressure after vitamin B-12, folic acid and combination treatments, associated with a significant improvement in augmentation index, a marker of arterial stiffness (135% ± 22% to 122% ± 21%, *p* < 0.001) (Figure 2) [65]. Vitamin B-12 supplementation either alone or in combination with folic acid, improved arterial stiffness, endothelial function and carotid IMT in northern rural Chinese, suggesting a potentially novel and affordable atherosclerosis prevention strategy in subjects with subnormal vitamin B-12 status.

Till *et al.* reported a significant decrease of carotid IMT in 26 nonvegetarian subjects at high risk of cerebral ischemia (baseline carotid IMT > 1.0 mm) after vitamin B supplementation for one year (1.50 ± 0.44 to 1.42 ± 0.48 mm, *p* = 0.034), compared with an increase (1.47 ± 0.57 to 1.54 ± 0.71 mm, *p* = 0.015) after placebo treatment, associated with a significant homocysteine reduction of 3.94 + 3.19 μmol/L after vitamin B supplementation but not placebo treatment [66]. However, in the nonvegetarian coronary or post-stroke (VITATOPS) cohorts with presumably normal vitamin B-12 status, vitamin B homocysteine-lowering supplementation induced no significant long term changes in rate of coronary restenosis, carotid IMT or brachial artery reactivity [67,68], which could explain the neutral results of a few large prospective vitamin B homocysteine-lowering supplementation studies on cardiovascular endpoints [69,70,71,72].

## 7. General Remarks

Vegetarians, in particular vegans in India and China, have a high prevalence of metabolic vitamin B-12 deficiency. Consumption of dairy or egg products together with high fruits, vegetables, grain and vitamin C intakes may partially alleviate the metabolic and vascular adverse effects, resulting in a more vascular-healthy environment with fewer coronary and stroke events. However, restriction or exclusion of all animal food, including dairy and egg products, in strict vegans may result in lower intake of vitamin B-12, and subsequently metabolic vitamin B-12 deficiency and adverse vascular surrogates (brachial FMD and carotid IMT). Vegetarian diets are protective for cardiovascular risk and events. Vegan diets are more protective compared with lacto-ovo-vegetarian diets for body mass index, and prevalence of diabetes mellitus, hypertension and hyperlipidemia. However, in cohorts with normal or relatively high salt intake and subnormal vitamin B12 status, such as in Chinese vegans, there is an adverse impact of vegan diets on atherosclerosis surrogates (arterial endothelial function and carotid intima-media thickness), which is potentially ameliorable by vitamin B-12 supplementation. Whether this benefit could be extended to other vegans with lower salt intake and better blood pressure profiles will remain to be confirmed. What needs to be tested is whether such early vascular changes in function and structure will eventually lead to more cardiovascular events [56], and whether improvement in vascular surrogates by vitamin B-12 supplementation will translate into a reduction in such events. Further studies, incorporating larger sample size are needed to dissect the mechanisms of improvement *i.e.*, whether the benefit if any, is due to independent vitamin B-12 effects, homocysteine-lowering and/or via reduction in blood pressure. For many reports on atherosclerosis surrogates and cardiovascular events, data on certain confounding factors including smoking, alcohol, physical activity and folate status were not entirely present or checked. Ideally these should be more systematically monitored in comparative studies in the future.

Early symptoms of vitamin B-12 deficiency are nonspecific (unusual fatiguability and digestion problems), and presenting hematological and neurological signs may be quite subtle. Hence vegetarians in particular vegans need to be advised to carefully plan their diets, and to monitor their plasma vitamin B-12 on a more regular basis, to facilitate early detection of low vitamin B-12 status, and if necessary to take vitamin B-12 fortified food, B-12 supplementation, or milk products [55,73]. In order to reap the full benefits of cardiovascular disease prevention in plant-based eating styles of vegan diets, individuals should maintain adequate vitamin B-12 status.

## 8. Conclusions

Metabolic vitamin B-12 deficiency is prevalent in vegetarians and, in particular, in vegans. Those subjects with normal or relatively high salt intake may be associated with unhealthy early vascular changes in function and structure, which have not been well documented in the past. In individuals with subnormal vitamin B-12 status, vitamin B-12 supplementation may significantly improve such vascular changes. Regular monitoring of vitamin B-12 profile may thus be beneficial for early detection and treatment of metabolic vitamin B-12 deficiency, and possibly prevention of atherosclerosis-related diseases.
